# Spouse-Appraised Memory Functioning Predicts Memory Decline Better Than Subjective Memory Complaints in Community Dwelling Older Adults at Genetic Risk for Alzheimer's Disease

**DOI:** 10.3389/fpsyt.2021.633102

**Published:** 2021-02-22

**Authors:** Youssef Bellaali, John L. Woodard, Bernard Hanseeuw, Adrian Ivanoiu

**Affiliations:** ^1^Institute of Neuroscience, Université Catholique de Louvain, Brussels, Belgium; ^2^Neurology Department, Saint Luc University Hospital, Université Catholique de Louvain, Brussels, Belgium; ^3^Psychology Department, Wayne State University, Detroit, MI, United States; ^4^Radiology Department, Massachusetts General Hospital, Harvard Medical School, Boston, MA, United States

**Keywords:** lack of awareness, subjective memory complaints, preclinical Alzheimer disease, memory impairments, relative's account, Apolipoprotein E, wisconsin longitudinal study

## Abstract

**Objective:** Alzheimer's disease (AD) begins with subtle memory decline, years before dementia onset. The presence of subjective memory complaints (SMC) has been proposed as a marker of preclinical AD. However, recent evidence has demonstrated early and progressive loss of awareness of memory difficulties in non-demented older adults harboring AD pathology. We investigated the respective contributions of SMC and spouse-appraised memory functioning (SAM) to predict memory decline in a large cohort of community dwelling older adults.

**Methods:** The Wisconsin Longitudinal Study collected cognitive data from a community-based cohort of 3,583 participants in both 2005 and 2011. The participant and the participant's spouse were each asked to rate the participant's memory functioning using a Likert scale. We predicted change in objective episodic memory with models including baseline SMC, baseline SAM, or both SMC and SAM. We also evaluated an awareness index (SMC minus SAM). We then tested the interaction between Apolipoprotein E (APOE ε4) carrier status and SMC/SAM to evaluate whether the effects were driven by individuals at-risk for AD pathology.

**Results:** In separate models, SMC (−0.081 ± 0.036, *p* = 0.025) and SAM (−0.084 ± 0.278, *p* = 0.003) were both associated with memory decline over ~6 years. However, the AI was not significantly associated with memory decline (0.031 ± 0.024, *p* = 0.19). When both predictors were included in the same model, SAM (−0.074 ± 0.03, *p* = 0.0092) was associated with memory decline, while SMC was not significant (−0.061 ± 0.04, *p* = 0.99). The association between SAM and memory decline was stronger in the APOE ε4 carriers than in the non-carriers (APOE-by-SAM interaction: *F* = 6.07; *p* = 0.002), and follow up analyses revealed that SAM was particularly predictive of decline only for APOE ε4 carriers. The association between SMC and memory decline was independent of APOE ε4 carrier status (APOE-by-SMC interaction: *F* = 2.29; *p* = 0.13).

**Conclusions:** Spouse-appraised memory functioning was more predictive of memory decline than SMC or an awareness index, particularly in APOE ε4 carriers, who are at increased risk for AD pathology.

## Introduction

Longitudinal studies of clinically unimpaired older individuals suggest subtle memory change is evident as early as 12 years before the diagnosis of Alzheimer's disease (AD) dementia (1). Because cross-sectional neuropsychological evaluation long remains in the normal range, subjective memory complaints (SMC) have been suggested as a possible harbinger of preclinical AD (2–4). However, anosognosia –a symptom defined as the lack of awareness (LOA) of one's own cognitive deficits– is well-documented in AD dementia (5, 6), and it may already be observed in the preclinical and prodromal stages of AD (7, 8).

Awareness is often operationalized by subtracting the degree or level of complaints expressed by a relative, most often the spouse, (i.e., SAM for spouse-appraised memory functioning) from the participant's degree or level of complaints (SMC). An awareness index of zero, therefore, suggests that the participant's insight into his/her level of memory functioning matches the spouse's observations. When the index is positive, the participant may be reporting more memory concerns (or may be more hyperaware) than the spouse's observations would suggest. Finally, when it is negative, the participant may be underreporting memory difficulties relative to the spouse's report, suggesting the possibility of at least partial unawareness of his/her memory abilities.

Previous studies have shown that AD biomarker-positive patients with mild cognitive impairment (MCI) already show LOA (9–11), whereas pre-symptomatic amyloid positive individuals may initially be hyperaware and may express SMC (7, 9, 12, 13). However, it is not clear whether some degree of LOA may already be present during the preclinical, asymptomatic stage of AD, thereby decreasing the value of SMC for predicting subsequent memory decline. To our knowledge, a study directly comparing SAM and SMC in clinically unimpaired older individuals at risk of developing AD has yet to be performed.

To this end, we investigated a unique community-based cohort of clinically unimpaired older adults from the Wisconsin Longitudinal Study (WLS) (14). We used SAM, SMC, and an awareness index to predict changes in memory performance over the next 6 years. We hypothesized that because of incipient LOA, SAM would be more predictive than SMC of memory decline.

## Methods

### Participants

The Wisconsin Longitudinal Study (WLS) includes more than 10,000 high school graduates from the State of Wisconsin. Most participants have been followed longitudinally since their graduation in 1957 (14). The data were collected via various media (interview, telephone and written questionnaire) at several time points, most recently in 2005 and 2011. The WLS provides neuropsychological and functional assessment data in addition to genetic data, including Apolipoprotein E (APOE), whose ε4 allele is a well-known genetic risk factor for developing sporadic AD (14). From this sample, 3,583 participants were selected based on the availability of the needed data. Genetic data were available in a subgroup of 2,605 subjects. A data sharing agreement and ethical approval was obtained for data analysis at UCLouvain (2018/07JUI/245).

### Subjective Memory Complaint, Spouse Appraisal of Memory and Awareness Scores

In the 2005, participants and their spouses each completed surveys rating the level of the participant's memory functioning. Participants were asked, “During the past 4 weeks, how would you describe your ability to remember things?” The possible responses were: 1-remember most things, 2-somewhat forgetful, 3-very forgetful, and 4-unable to remember anything, which collectively reflected the severity of the participants' SMC. For the spouse appraisal of memory, Spouses were asked, “How would you rate your spouse's memory at the present time?” The possible responses were: 1-Poor, 2-Fair, 3-Good and 4-Very Good. Because the two surveys were on different scales and in reverse order, we recoded the spouse's report (4 became 1, etc.). We then created an awareness index (AI) by computing the difference between each participant's self-evaluation and the assessment made by the participant's spouse, which ranged from −3 to +3. Increasingly negative scores reflect more underestimation of memory difficulty, while increasingly positive scores suggest overestimation of memory difficulties, relative to what the spouse reported. This method of assessing awareness using a caregiver appraisal of the participant's memory functioning and the participant's self-evaluation of his/her own memory functioning was inspired by the Starkstein Anosognosia Questionnaire for Dementia (AQ-D) (15).

### Objective Memory Performance

Objective memory performance was measured both in 2005 and 2011 using a composite score based on the single-trial immediate and delayed recall of a 10-word list (c-memory). Raw scores for both the 2005 and 2011 evaluations were standardized to obtain *z*-scores using the mean and standard deviation of the whole group within each time point. Finally, we calculated an arithmetic mean of the immediate and delayed *z*-scores to obtain the following scores: c-memory 2005 (baseline) and c-memory 2011 (follow-up).

### Statistical Analysis

We performed a Spearman correlation between SAM and SMC baseline data (2005), to evaluate the association between the spouse's assessment of the participant's level of memory functioning and the participant's own subjective self-evaluation of his or her memory functioning.

In order to assess the association between the subjects' and spouses' complaints and the objective memory scores at baseline we performed a multiple regression analysis using c-memory 2005 as the dependent variable and SAM, SMC, and AI as predictors by taking into account the effects of age, sex and education.

To evaluate the prediction of memory decline from the subjective complaints we used the c-memory scores to assess memory decline from 2005 to 2011. We predicted the 2011 scores from the 2005 scores using bivariate regression, and we saved the standardized residual for each participant as their residualized change scores. Multiple regressions evaluated the association between memory decline (residualized change scores) and SMC, SAM and AI by taking into account the effects of age, sex and education.

We performed a *t*-test to assess differences between APOE ε4 carriers and non-carriers for baseline memory performance and memory decline.

Finally, we conducted two-way ANOVAs to test for an interaction effect between APOE ε4 carrier status and the three rating scores (i.e., SMC, SAM, and AI) when predicting memory decline.

The analyses were performed using MedCalc for Windows V.19.0.5 (MedCalc Software, Ostend, Belgium) (16).

## Results

### Preliminary Analysis of Baseline 2005 Demographics, APOE Status and the Awareness Measures (SMC, SAM, and AI)

As shown in Table 1, a majority of participants (76.7 %) declared they “remember most things” (level 1), but an important minority (23.2%) reported some forgetfulness, most often a mild degree (level 2; 22.6%). Overall, spouses were more critical of participants' memory functioning than the participants themselves. For example, 29.5% of the spouses reported the participant's memory function to be at level 2 or higher (suggesting greater forgetfulness) compared to only 23.2 % of the participants.

**Table 1:** Self and spouse memory evaluation in 2005.

		**Spouse evaluation**				**Total**
		**Very Good**	**Good**	**Fair**	**Poor**	
Self-evaluation	Remember most things	2,078	565	96	11	2,750 (76.7%)
	Somewhat forgetful	447	281	72	11	811 (22.6%)
	Very forgetful	4	7	7	3	21 (0.6%)
	Unable to remember anything	0	0	1	0	1 (<0.1%)
Total		2,529 (70.6%)	853 (23.8%)	176 (4.9%)	25 (0.7%)	3,583

The correlation between participants and spouses was statistically significant (Spearman rho = 0.209; *p* < 0.0001), although the magnitude of this relationship was relatively modest, as the participants' self-reports explained only 4.3% of the variance in the spouse report.

**Figure 1:**
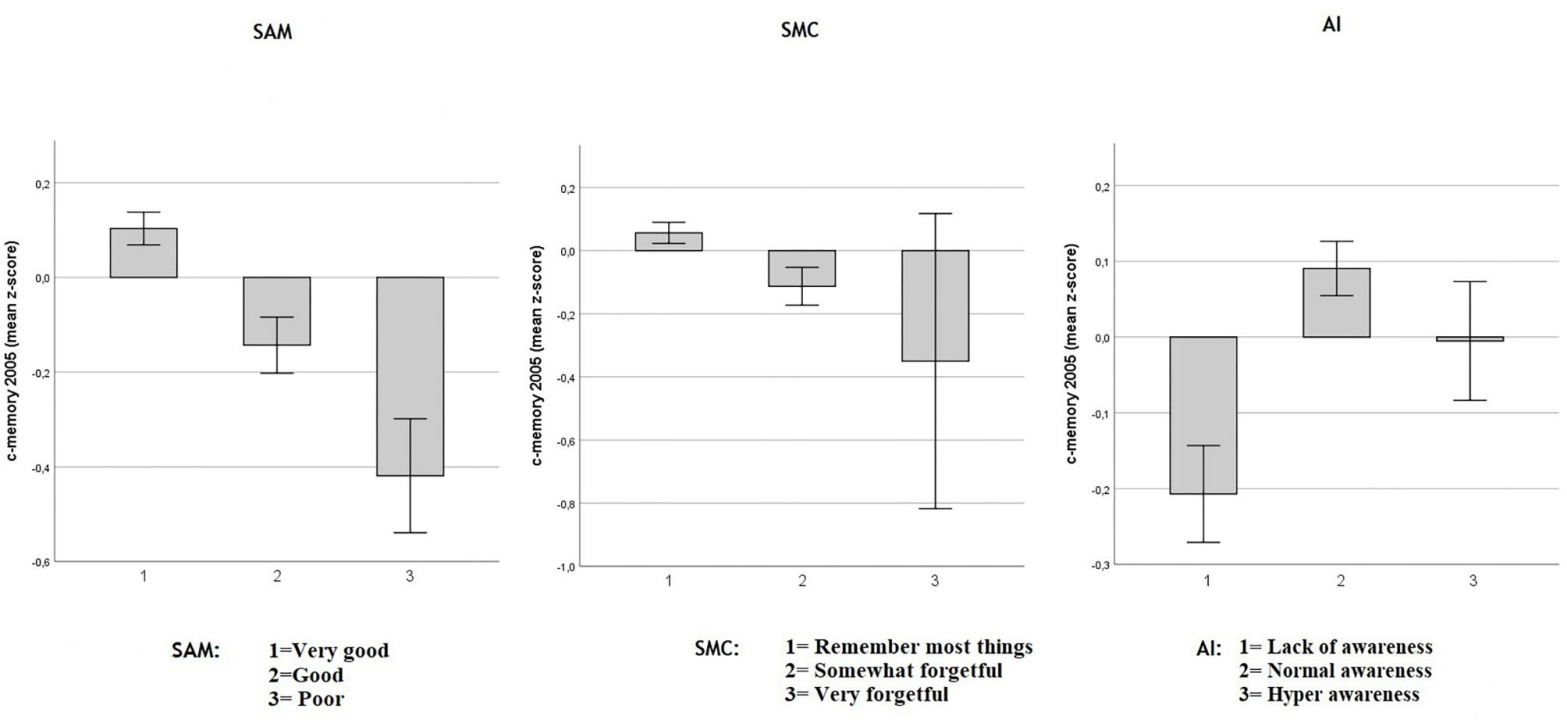
Baseline objective memory in 2005 in SAM, SMC, and AI. Values reflect baseline (2005) group means (with 95% CIs) for c-memory 2005 (mean of the immediate and delayed memory *z*-scores). Respectively in three multiple regression models including age, sex and education Spouse-Appraised Memory functioning (SAM) (*p* < 0.0001), Subjective Memory Complaint score (SMC) (*p* < 0.0001) and Awareness Index (AI) (*p* < 0.0001) were correlated with baseline c-memory. When SAM and SMC were included in the same model, adjusted for age, sex and education, both SMC (*p* = 0.019) and SAM (*p* < 0.0001) were independently associated with baseline c-memory.

**Figure 2:**
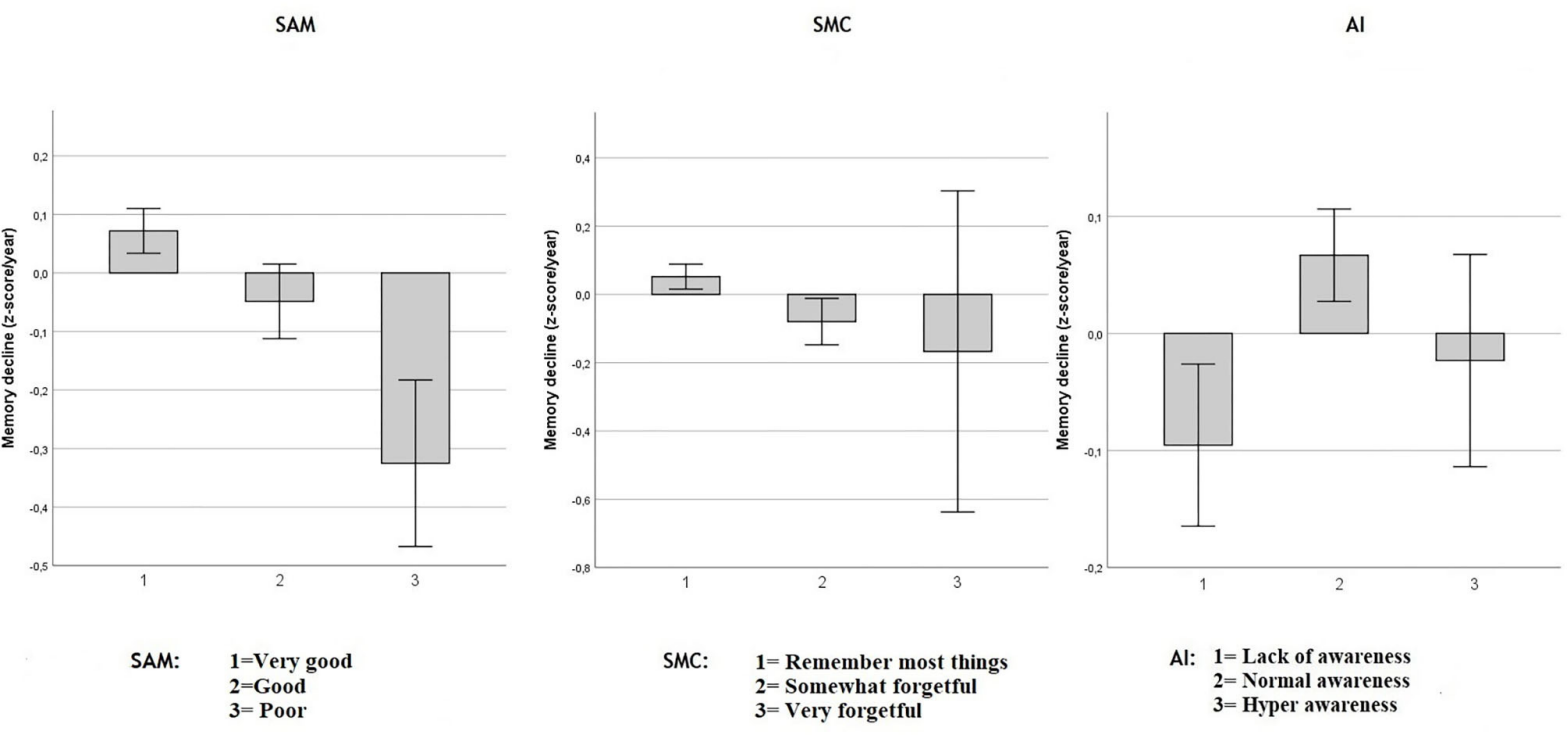
Objective memory decline from 2005 to 2011 in SAM, SMC, and AI. Values reflect group means (with 95% CI) for residualized memory change between 2005 and 2011 in *z*-score/year. Respectively in three independent multiple regression model including age, sex and education: Spouse-Appraised Memory functioning (SAM) (*p* = 0.003) and Subjective Memory Complaints (SMC) (*p* = 0.025) were statistically associated with memory decline over ~6 years. AI was not significantly associated with memory decline (*p* = 0.195). When SAM and SMC were included in the same model, adjusted for age, sex and education, only SAM was statistically associated with memory decline (*p* = 0.0092).

Because no participants and very few spouses endorsed deficient functioning at level 4 (Table 1), ratings at level 3 and 4 were pooled for further analysis (SMC score: 1-remember most things, 2-somewhat forgetful, 3-very forgetful; SAM score: 1-very good, 2-good, 3-poor). It is important to note that there was considerable variability in SMC ratings for the “very forgetful” level (see also Figures 1, 2, **4** below), presumably due to relatively few participants who considered their own memory functioning to be problematic. However, spouse ratings at all three levels were much less variable, suggesting the possibility of greater reliability of spouse-appraised memory relative to the participant's own SMCs. The calculated AI showed: −3 (0.3%); −2 (3%); −1 (17.9%); 0 (66%): 1 (12.7%) 2 (0.1%) and 3 (0%). Subsequently, we pooled the scores into three groups: negative scores as “Lack of awareness” (LOA), the null score as “Normal awareness” (NA) and the positive scores as “Hyper awareness” (HA).

The demographics and APOE status for the whole sample and all the subgroups (SMC, SAM, and AI) are shown in Table 2. For the whole sample, 684 participants were ε4 carriers, 1,921 were ε4 non-carriers, and 978 had unknown genotype (subpopulation with available APOE genotyping = 2605). In the ε4 carrier group, 46 participants were homozygous for the ε4 allele while 638 were heterozygous.

**Table 2:** Baseline sample demographics.

	**All (3,583)**	**SMC = 1 (2,750)**	**SMC = 2 (811)**	**SMC = 3 (22)**
Mean age in 2005 (years)	63.8	63.8	63.9	64.7
Sex (% female)	45.8%	46.4%	44.5%	27.3%
Education (years)	14.2	14.4	13.8	12.9
APOE4 carriers (%) (subpopulation *n* = 2,605)	26.1%	25.3%	28.7%	50%
	**All** **(3,583)**	**SAM = 1** **(2,529)**	**SAM = 2** **(853)**	**SAM = 3** **(201)**
Mean age in 2005 (years)	63.8	63.7	64.0	64.3
Sex (% female)	45.8%	48.8%	40.4%	31.3%
Education (years)	14.2	14.5	13.6	13.3
APOE4 carriers (%) (subpopulation *n* = 2,605)	26.1%	25.4%	28.8%	26.2%
	**All** **(3,583)**	**AI = LOA = −3,−2,−1** **(758)**	**AI = NA = 0** **(2,366)**	**AI = HA = +1,+2,+3** **(459)**
Mean age in 2005 (years)	63.8	64.1	63.7	63.8
Sex (% female)	45.8%	39.4%	47%	50.3%
Education (years)	14.2	13.6	14.4	14.2
APOE4 carriers (%) (subpopulation *n* = 2,605)	26.1%	26.0 %	26.2%	27.1%

*SMC 1, remember most things; SMC 2, somewhat forgetful; SMC3, very forgetful. SAM 1, Very good; SAM 2, Good; SAM 3, Poor. For AI, LOA, lack of awareness; NA, normal awareness; HA, Hyper awareness*.

### Objective Memory Performance According to SMC, SAM, and AI

#### Baseline 2005

As shown in Figure 1, decreased objective memory performance was modestly associated with increasing SMC in a multiple regression model including age, sex and education (adjusted-R^2^ = 0.10; unstandardized SMC coefficient: −0.118, SE = 0.032, *p* < 0.0001) and with increasing SAM (adjusted-R^2^ = 0.11; unstandardized SAM coefficient: −0.165, SE = 0.025, *p* < 0.0001). The AI highlighted that participants who overestimated their memory had lower objective memory performance in a multiple regression model including age, sex and education (adjusted-R^2^ = 0.104; unstandardized AI coefficient = 0.077, SE = 0.021; *p* < 0.0001). Both SMC and SAM independently predicted the objective c-memory composite score when both were included in a multiple regression model including sex age and education: (adjusted-R^2^ = 0.11; unstandardized SMC coefficient: −0.077, SE = 0.033, *p* = 0.019 and unstandardized SAM coefficient: −0.153, SE = 0.025, *p* < 0.0001), indicating that SAM was associated more strongly with objective memory than was SMC at baseline. Because AI is a composite index based on the discrepancy between SAM and SMC, a multiple regression model including these three predictors was not performed as it would have created a perfect multicollinearity between AI and the SMC and SAM predictors.

#### Memory Decline From 2005 to 2011

As shown in Figure 2, in separate models, including age, sex and education, both SAM (adjusted-R^2^ = 0.068; unstandardized coefficient: −0.084, SE = 0.278, *p* = 0.003) and SMC (adjusted-R^2^ = 0.067; unstandardized coefficient: −0.081, SE = 0.036, *p* = 0.025) were associated with memory decline over ~6 years. However, the AI was not significantly associated with memory decline (adjusted-R^2^ = 0.066; unstandardized AI coefficient: 0.031, SE = 0.024, *p* = 0.195).

When both SAM and SMC were included in the same multiple regression model, including age, sex and education, only SAM was associated with memory decline (adjusted-R^2^ = 0.069; unstandardized SAM coefficient = −0.074, SE = 0.03, *p* < 0.0092) while SMC was not (unstandardized SMC coefficient = −0.061, SE = 0.04, *p* = 0.991). Adding the objective memory score from 2005 to the model did not modify this result. That is, SAM predicted subsequent memory decline above and beyond baseline objective memory performance and SMC. Because of the perfect multicollinearity between AI and the SAM and SMC predictors, a model including all three predictors was not performed.

## Objective Baseline Memory Performance and Memory Decline According to the APOE Carrier Status

**Figure 3:**
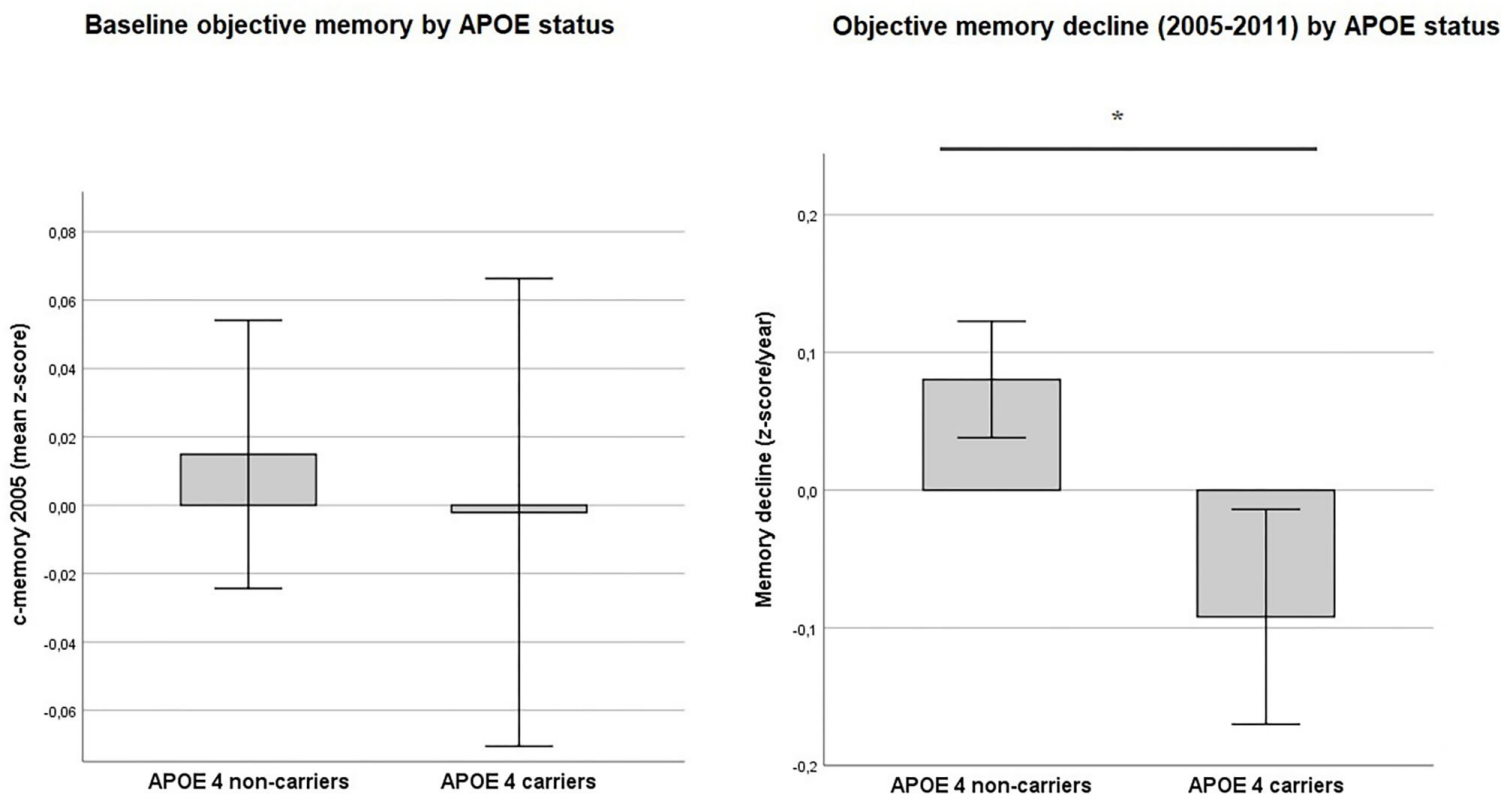
Objective memory by APOE status. Values reflect group means (with 95% CIs) for baseline objective memory in 2005 (c-memory 2005) and for residualized memory change between 2005 and 2011 (memory decline). No statistically significant difference was found between APOE ε4 carriers and non-carriers for baseline c-memory 2005) (*p* = 0.08). In contrast, ε4 carriers showed a greater memory decline from 2005 to 2011 (memory decline) than non-carriers (*p* < 0.001). *Intergroup comparison is significant at *p* < 0.05.

Baseline memory performance in 2005 was not statistically different between e4 carriers and non-carriers. *T*-test [*t*_(2603)_ = −0.43, *p* = 0.08] (see also Figure 3). However, for memory decline e4 carrier status was shown to be significant (Figure 3) *T*-test: [*t*_(2603)_ = −3.99, *p* < 0.001].

## Interaction Effect Between SMC, SAM, AI, and APOE Status on Memory Decline

**Figure 4:**
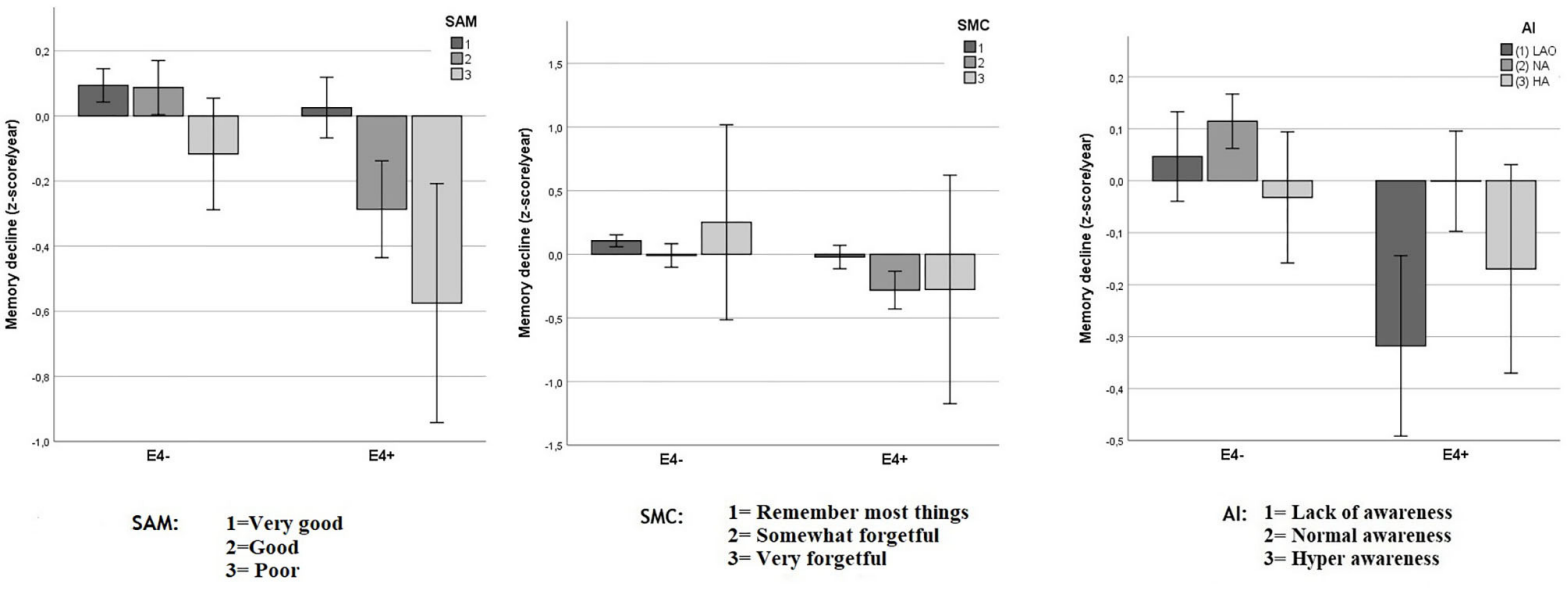
Objective memory decline from 2005 to 2011 in SAM, SMC, and AI as function of APOE status. Values reflect group means for residualized change scores in *z*-score/year (with 95% CIs) that indicate memory decline between 2005 and 2011. A significant interaction was observed between APOE status and Spouse-Appraised Memory functioning (SAM) (*p* = 0.002) but not between APOE and Subjective Memory Complaints (SMC) (*p* = 0.265) or APOE and the awareness index (AI) (*p* = 0.065). “E4+” = APOE ε4 carriers, “E4–” = APOE ε4 non-carriers.

As shown in Figure 4, the relationship between SAM and the memory decline appeared to be mediated by the APOE status, whereas this is not the case for SMC and AI.

We tested for the presence of an interaction effect between SMC, SAM, AI, and APOE status on memory decline using three separate factorial ANOVAs, each with two factors.

When SMC was considered, the analysis showed a trend for an ε4 carrier status main effect [*F*_(1, 2599)_ = 3.52; *p* = 0.061], a significant main effect for SMC [*F*_(2, 2599)_ = 7.09; *p* = 0.001], and no significant interaction effect [*F*_(2, 2599)_ = 1.33; *p* = 0.265]. Because there were very few participants with ratings of level 3 in both APOE subgroups (eight participants in each group), we pooled levels 2 and 3. After doing so, the ε4 carrier status main effect was statistically significant [*F*_(1, 2601)_ = 16.9; *p* < 0.001], as was the SMC main effect [*F*_(1, 2601)_ = 14.2; *p* < 0.001]. However, there still was no significant interaction effect [*F*_(1, 2601)_ = 2.289; *p* = 0.130].

The same analysis performed with the spouses' ratings (SAM) showed a main effect of ε4 carrier status [*F*_(1, 2599)_ = 18.85; *p* < 0.001], a main effect for SAM [*F*_(2, 2599)_ = 12.6; *p* < 0.001], and a significant interaction effect [*F*_(2, 2599)_ = 6.07; *p* = 0.002, Figure 4). When level 2 and 3 were pooled the main effect for ε4 carrier status remained statistically significant [*F*_(1, 2601)_ = 23.8; *p* < 0.001], as did the SAM main effect [*F*_(1, 2601)_ = 19.3; *p* < 0.001] and the interaction effect between ε4 carrier status and SAM [*F*_(1, 2601)_ = 11.6; *p* = 0.001]. Decomposition of this significant interaction revealed that for non-carriers, there were no statistically significant differences in memory decline between the two SAM rating categories [*F*_(1, 1919)_ = 0.9, *p* = 0.3]. In contrast, for carriers, memory decline was greater for persons with larger SAM ratings [*F*_(1, 682)_ = 18.6, *p* < 0.0001].

When AI was used as independent variable with ε4 carrier status, the analysis showed only a non-significant trend for the interaction [*F*_(2, 2599)_ = 2.74; *p* = 0.065], while main effects of both ε4 carrier status [*F*_(1, 2599)_ = 15.19; *p* < 0.001] and AI [*F*_(2, 2599)_ = 7,96; *p* < 0.001] remained significant.

## Discussion

Our results demonstrate that SAM is a particularly strong non-biological predictive index of cognitive decline in a preclinical population, compared to other markers such as SMC or AI that are commonly used with patients with neurodegenerative disease, and it is particularly predictive of decline for APOE ε4 carriers.

APOE ε4, a well-known surrogate marker of the presence of amyloid deposits in the brain, was the most powerful predictor of memory decline in our sample. This finding concurs with the vast literature supporting a specific relationship between this genetic polymorphism and AD risk (17, 18), where memory impairment is the earliest and most salient cognitive deficit (19). Interestingly, APOE ε4 carriers and non-carriers were similar on baseline memory scores, suggesting the absence of a relationship between cognitive performance and the presence of the APOE ε4 allele at the sample's mean age of 64 in 2005. Nearly one-quarter (21%) of community dwelling older adults in our sample rated their memory functioning as being better than their spouse considered it to be. In contrast, 13% showed an opposite pattern of increased SMC relative to spousal ratings (i.e., the participant reported more memory difficulty than the spouse reported).

Our analyses also suggested APOE carrier status was independent of SMC severity ratings when predicting cognitive decline. In contrast, when the SAM index was considered with carrier status, greater SAM severity ratings were associated with cognitive decline only in participants carrying the APOE ε4 risk allele. These results suggest that SAM predicts cognitive evolution in a population at risk of developing AD at a preclinical stage.

These two preclinical predictors, APOE and SAM, are easily obtained, inexpensive, and ultimately not invasive. Moreover, the synergistic use of these two risk markers makes it possible to identify a group at the greatest risk for memory decline, several years in advance.

For almost three decades, it has been very clearly demonstrated in the literature that a large proportion of patients with AD dementia experience anosognosia. The percentage of anosognosic AD patients increases throughout the disease course. At the severe stage, up to 80% of patients show some degree of anosognosia (9). Indeed, across all stages of AD severity, all past studies demonstrate a clear loss of awareness in a substantial proportion of AD patients (6, 17–19).

The presence of unawareness of deficits in MCI and during the preclinical stage has been less consistently demonstrated. Several studies have highlighted that among persons with MCI, anosognosia may be present in a less obvious fashion than in AD, but it is nevertheless already well-established in high-amyloid MCI (7, 8). On the other hand, it has also been demonstrated that hyperawareness of deficits manifested by SMCs can predict subsequent cognitive decline during the preclinical stage and in persons with MCI (2, 3, 20–22). Looking at the extant body of literature, a way of reconciling these apparently diverse results is to conceptualize an evolution of awareness to unawareness during the pathophysiological progression of AD. During preclinical stages, patients may show increased awareness of cognitive changes. Subsequently, as the disease course progresses to MCI, anosognosia becomes increasingly prevalent until it reaches clinically obvious levels in AD.

Unexpectedly, our study suggests that persons at the pre-dementia stage are, in fact, not hyper-vigilant. Our results revealed that while each participant's own self-rating only weakly predicted his or her cognitive decline, the spouse's assessment did so more effectively. Therefore, our findings are at odds with those of several past studies that have reported the preclinical presence of SMC and implicating an early hypervigilance toward cognitive changes.

Given the discrepancies in results between our study and the conclusions of others, it is important to point out possible study differences that could account for this discrepancy. First, these previous studies in SMC did not take into account SAM. Consistent with these studies, we observed a weak association between SMC and subsequent decline. Moreover, the study population in the WLS is younger (average age of participants 64 years) than other studies showing a relationship between SMC and cognitive decline [mean age 72 years (20) and 69 years (21) respectively]. As the prevalence of AD increases exponentially with age, it is probable that the memory deficit would have been greater in the previously studied cohorts. This difference could have played out both ways on SMC, making the participants more willing to report memory concerns because of a greater memory deficit (increasing SMC), but at the same time, the number of participants showing an onset of anosognosia due to incipient AD (decreasing SMC) may also have increased. This combination could result in a complex relationship that is not directly comparable to our cohort. Most importantly, the absence of SAM evaluation in previous studies does not allow a meaningful comparison. However, despite having a younger cohort with, arguably, better memory functioning than in previous studies, SAM was strongly associated with cognitive decline, whereas SMC was clearly less so. It is well-known that the prevalence of AD increases with age. However, despite having a younger cohort characterized only for ε4 and not for amyloid, SAM was strongly associated with cognitive decline, whereas SMC was clearly less so taking into account SAM in future studies may help identify earlier clinically normal older adults who may be at risk of cognitive decline in the near future.

Major strengths of our study include the very large sample size and relative homogeneity of participant ages at both measurement periods. These factors enabled us to demonstrate the power of SAM for predicting cognitive decline that was independent of SMC. Indeed, given the very modest correlation between SAM and SMC the two indexes appear to be evaluating different dimensions of awareness. The major limitation of our study lies in its retrospective nature, not permitting us to identify prospective risk factors that would be sensitive to cognitive decline. In addition, only one measure of each participant's memory self-evaluation and of the spouse's evaluation of each participant's memory in 2005 was available. This measure was not obtained in 2011, making it difficult to determine whether either evaluation may have changed over time due to disease progression or other factors. The data from this study were also obtained from participants with spouses, and it is uncertain whether participants without spouses may have responded in the same manner. Finally, participants who responded to the cognitive survey were included in our study. The participants who did not respond were excluded, leading to possible selection bias in our analysis because of potential differences between the group who endorsed the cognitive survey and the persons who did not respond. To our knowledge, this study is the first to show the value of SAM as an early marker of cognitive decline in community dwelling older adults, in combination with genetic risk for AD. This study suggests that evaluation of memory functioning by close informants, such as family members or regular caregivers, may be more effective than an awareness index or SMC for identifying at an early stage individuals at-risk for cognitive decline, particularly among carriers of the APOE ε4 allele.

## Data Availability Statement

The data analyzed in this study is subject to the following licenses/restrictions: See WLS restrictions on https://www.ssc.wisc.edu/wlsresearch/. Requests to access these datasets should be directed to wls@ssc.wisc.edu.

## Author Contributions

YB, BH, JW, and AI contributed to the design of the study, to the analysis of the results and to the writing of the manuscript. All authors contributed to the article and approved the submitted version.

## Conflict of Interest

The authors declare that the research was conducted in the absence of any commercial or financial relationships that could be construed as a potential conflict of interest.
